# Supporting the global reduction of cervical cancer: challenges in colposcopy practice and training

**DOI:** 10.3389/fonc.2025.1592050

**Published:** 2025-09-08

**Authors:** Takuma Fujii, Yusuke Taira, Grainne Flannelly, Haruki Nishizawa, Yoshimichi Tanaka

**Affiliations:** ^1^ Department of Gynecology, School of Medicine, Fujita Health University, Toyoake, Aichi, Japan; ^2^ Laboratory For Health, Tokyo Health Service Association, Tokyo, Japan; ^3^ Department of Obstetrics and Gynecology, School of Medicine, Fujita Health University, Toyoake, Aichi, Japan; ^4^ Department of Obstetrics and Gynecology, Ryukyu University, School of Medicine, Ginowan, Okinawa, Japan; ^5^ School of Medicine, University College Dublin, Dublin, Ireland; ^6^ Department of Obstetrics and Gynecology, School of Medicine, Osaka Medical and Pharmaceutical University, Takatsuki, Osaka, Japan

**Keywords:** colposcopy, cervical cancer, screening, training, practice, cervical intraepithelial neoplasia, human papillomavirus, quality assurance

## Abstract

Colposcopy is an important element in the global challenge of reducing cervical cancer incidence. However, there are issues with expanding its use globally, including resources, training of colposcopists and quality assurance. This narrative review explores the role of colposcopists and the challenges of ensuring that practice and training are fit for purpose. The review found that colposcopy and colposcopists play three roles in managing cervical cancer: in diagnosis and interventions; communicating with patients; and supporting public education. It also found that colposcopy practices are evolving globally, alongside regional and national variations in vaccination, screening and treatment practices, creating important challenges. Many countries have introduced quality indicators or standards, but studies show significant variations from these in practice. Training of colposcopists also varies across regions. Some developed countries have defined curricula, but developing countries are more reliant on distance learning courses provided by international bodies. The review identified several ways to address these challenges, including setting standards for either practice or training, and training healthcare professionals from different backgrounds as colposcopists. New technologies such as artificial intelligence could also help. The review also identified some gaps in the literature, offering potential for further research. These include developing a consensus on the training needs in particular areas, and exploring how global or regional standards are applied at local levels, and how training for soft skills can best be delivered to colposcopists. It is considered essential that educational curricula should be tailored to the specific circumstances of each country or region.

## 1 Introduction

Cervical cancer remains a major global public health challenge and is the fourth most common cancer among women, with approximately 600,000 new cases and more than 300,000 deaths reported each year (1). While the burden is particularly heavy in low- and middle-income countries—accounting for 94% of cervical cancer-related deaths (2)—Japan represents a unique case among high-income nations. Despite having a well-established healthcare infrastructure and economic prosperity, Japan continues to report relatively high cervical cancer incidence and mortality, with approximately 10,000 new diagnoses (3) and 2,800 deaths annually (4). These unfavorable outcomes are thought to result in part from persistently low HPV vaccination rates over the years, limited screening participation, and systemic weaknesses in the screening program, including inadequate call-recall systems.

Persistent infection with high-risk types of human papillomavirus (HPV) is the primary cause of cervical cancer (5, 6). To address this global health threat, the World Health Organization (WHO) launched the Global Strategy to Eliminate Cervical Cancer as a Public Health Problem, establishing the “90-70-90” targets for 2030: 90% of girls fully vaccinated against HPV, 70% of women screened at least once in their lifetime, and 90% of those diagnosed with cervical disease receiving appropriate treatment (7). Although HPV testing and other molecular technologies are reshaping cervical cancer screening strategies, colposcopy remains a critical diagnostic tool for the evaluation and management of screen-positive women. It plays a central role in assessing the severity and extent of cervical lesions and guiding decisions about biopsy or treatment.

Importantly, colposcopy is not merely a technical procedure. The process involves close patient interaction, including explaining abnormal results, discussing diagnostic and treatment options, and providing emotional support. Therefore, colposcopists are required to have not only procedural proficiency but also strong communication and counseling skills. These soft skills are essential for enhancing patient understanding, reducing anxiety, and fostering trust—factors that greatly influence adherence to follow-up and treatment (8, 9). Nevertheless, these non-technical competencies are often underemphasized in traditional training programs (10), which typically focus on diagnostic accuracy, image interpretation, and procedural techniques. As a result, many colposcopists may be underprepared to manage the interpersonal and ethical complexities encountered in real-world clinical practice. Furthermore, while previous literature has addressed the technical aspects of colposcopy, few reviews have comprehensively examined the broader roles of colposcopists, such as patient communication, psychological support, and public education. This gap is particularly notable in countries like Japan, where systemic challenges in cervical cancer prevention persist despite access to advanced healthcare. This review seeks to address that gap by exploring both the clinical and non-clinical dimensions of colposcopy, with the aim of rethinking how colposcopy practice and training should evolve in the modern era.

## 2 Roles and practices of colposcopists

Colposcopists vary widely in their educational and professional background, training and practice experience. Their professional background varies in particular by region or country. For example, in Japan, any licensed physician can perform colposcopy, but nurses do not do so. In some other countries, specially trained nurses routinely perform colposcopy [see, for example (11)]. Colposcopists and colposcopy services play a role in three key areas of cervical cancer prevention, detection and management. The first is during the diagnostic process and in interventions and treatment. The second is in communicating effectively with patients, both during interventions and in reassuring them, for example, to encourage them to attend follow-up appointments. The third is a more public health-based role, taking a stakeholder-led role in public education about cervical cancer screening and vaccination, as a specialist in cervical cancer and its precursor lesions.

### 2.1 Diagnostic processes and interventions

Colposcopists are required to master a theoretically based diagnostic process to guide patients toward the most suitable treatments. Various governing bodies and membership organizations have set out curricula for training and the knowledge and skills required by colposcopists [see, for example (12)]. **Table 1** summarizes key national and regional training programs and certification requirements.

**Table 1:** Examples of some national and international training and certification requirements for colposcopists.

Location	Britain	USA	Europe	Australia and New Zealand	Spain
Accrediting/ training body	British Society for Colposcopy and Cervical Pathology (BSCCP)	American Society for Colposcopy and Cervical Pathology (ASCCP)	European Federation of Colposcopy (EFC)	Royal Australian and New Zealand College of Obstetricians and Gynaecologists (RANZCOG)	Asociación Española de Patología Cervical y Colposcopia (AEPCC)
Details of training/education program	Training, objective structured clinical examination (OSCE), written exams, supervised clinical practice, participation in multi-disciplinary team (MDT) meetings, etc.	Three or four-days in-person or online (or on-demand) format	Acknowledging the flexibility of member countries, some items shared within the EFC, determined through the Delphi method by the member countries.	Online training program or other registered and approved training programs.	Webinars and regular courses.
Certification	Renewal every three years. Requires participation in continued medical education, attendance at conferences, audits, etc.	Not applicable; no certification	Certification standards aligned with EFC.	Defined certification and recertification requirements, including ongoing participation in continued medical education, plus evidence of practice.	Certification aligns with EFC standards. Valid for five years and automatically renewable upon meeting specific requirements.
url	https://www.bsccp.org.uk/colposcopist-training-accreditation	https://www.asccp.org/education/colposcopy-education-completion-exam-resources	https://efcolposcopy.eu/wp-content/uploads/2016/09/EFC_Certification_standars_2013.pdf	https://ranzcog.edu.au/womens-health/quality-assurance-programs/cquip/	https://www.aepcc.org/acreditacion-en-colposcopia-y-ptgi/

Accurate diagnosis demands a deep understanding of the development and progression of cervical tumors, the natural history of cervical tumors and HPV infection, cytology, histopathology, and cervical cancer screening guidelines. Studies have found that knowledge of cytology significantly improves the ability to interpret colposcopic images (13).

Real-world data from the United States show that the positive predictive value (PPV) of colposcopic impression for high-grade squamous intraepithelial lesions (HSIL) was only 56.2% for actual CIN2+ cases (14), indicating that the diagnostic accuracy of colposcopy may not be as high as commonly perceived. One possible explanation for this modest PPV is the limited number of biopsies taken during routine clinical practice. In particular, insufficient biopsy sampling by less experienced practitioners may contribute to the underdiagnosis of high-grade lesions. These findings underscore the importance of comprehensive training, including the ability to select the most suspicious areas for biopsy and to apply appropriate technique.

Studies in both developed and developing countries have shown that the diagnostic accuracy of colposcopy is comparable between gynecologists and nurse practitioners (15) and nurses and physicians (16). However, another study found that the diagnostic accuracy for HSIL or worse was 69.6% among junior colposcopists and 78.6% among senior colposcopists (17), highlighting the significant role of clinical experience. While such findings support the value of expertise, compensating for inexperience by taking excessive biopsies may lead to unnecessary harm (18) and should therefore be avoided.

In addition to diagnostic skills, colposcopists must be able to perform appropriate interventions. According to the European Federation for Colposcopy (12), those who complete basic training should be able to assess lesions and identify the most severe site, while more advanced procedures such as biopsies or surgical interventions are expected to be performed by practitioners with higher qualifications. The exact nature of interventions varies depending on regional systems and professional roles. For example, in the UK and Ireland, diagnostic biopsies are routinely included as part of the colposcopic evaluation when clinically indicated (19, 20). In Denmark, four-quadrant biopsies are recommended during diagnostic colposcopy (21). In Japan, patients with abnormal screening results are referred to general practitioners or directly to hospitals for further evaluation. When high-grade intraepithelial lesions are diagnosed, excisional procedures are typically performed by gynecologic oncologists, by gynecologists under their supervision, or independently by gynecologists. This reflects the historical context in which the Japanese Society for Colposcopy was merged with the Gynecologic Oncology Society (22).

### 2.2 Communication with patients

Colposcopists must minimize adverse events whenever possible and manage patient anxiety through effective communication (9). This includes the use of straightforward language and empathy to convey complex medical information and reduce concerns about diseases and testing. Effective communication skills are essential for delivering bad news, but also explaining the clinical findings and the likely management plan. This is particularly relevant for communications around HPV (10, 23). A qualitative study about the views of HPV-positive women on healthcare providers, and particularly patient–provider communication (24) highlighted the importance of the provider’s communication and counseling skills.

Women in that study (24) particularly identified the need to approach discussion of HPV with sensitivity, which makes sense in view of concerns raised in other studies about the anxiety associated with being labeled as HPV-positive (25, 26). Anxiety has also been seen among patients undergoing colposcopy (27). Patients often feel anxious about the procedure beforehand. However, a positive attitude among clinic staff can help alleviate this anxiety. Patients’ and providers’ choice of a facility for the procedure may be influenced by whether the patient can observe the colposcopy in real-time. The time between screening and follow-up examinations, and the time allocated for explanations and questions during the procedure are also crucial considerations (27). These considerations should be well understood by colposcopists, as it is important for them to recognize patients’ concerns.

In this context, soft skills—such as empathetic communication, active listening, and the ability to explain clinical findings in a clear and compassionate manner—are essential components of colposcopic practice. Despite their importance, these competencies are often underrepresented in formal colposcopy training programs. Some educational initiatives have begun to incorporate standardized patients (SPs), role-play, or OSCE-style formats to teach and assess communication skills in realistic clinical settings (28, 29). Furthermore, successful models from other specialties offer useful frameworks: for example, the SPIKES protocol in oncology provides a structured method for delivering bad news (30), while the CanMEDS framework emphasizes communication and professionalism as core physician competencies (31). In family medicine, the Calgary–Cambridge model provides structured guidance for patient-centered consultation (32). Adapting such models could enhance the quality and consistency of soft skill development within colposcopy training.

### 2.3 Public education on HPV vaccination and screening

Colposcopists can also play a role in public education on the importance of cervical cancer prevention and screening. This might include advising on the HPV vaccine, encouraging screening in eligible individuals, and urging follow-up for those who need further examination based on screening results. Education in colposcopy clinics has been shown to be effective for all of these. For example, a study in the USA found that a tablet-based education program in a colposcopy clinic was helpful in increasing patient knowledge about cervical cancer and HPV (33). After the program, more participants also said they would recommend HPV vaccination for a child in their family. This type of education program can therefore support the WHO’s global target of 90% HPV vaccination coverage and 70% of women screened, highlighting the potential impact of colposcopy clinics in supporting these goals.

This education is, however, limited to women referred to colposcopy services, which is likely to be a very small percentage of the eligible population. In the UK in 2022–23, for example, the proportion of women referred to colposcopy services was approximately 7% of those screened and 5% of those eligible, and only 73% of first appointments were actually attended (34). The percentage of appointments attended may also be significantly lower in other settings. One study in Mexico City found that only 57% of participants attended their first colposcopy appointment (35). The provision of information to women attending colposcopy clinics is therefore only ever likely to play a small role in overall public education on HPV vaccination and screening.

## 3 Recent evolution of cervical cancer prevention and treatment, and colposcopy practice

Cervical cancer prevention and treatment are evolving rapidly. Primary prevention in many countries now focuses on vaccination programs against HPV. This has reduced the number of infections and therefore lowered the risk of cervical cancer (5, 6). Secondary prevention comprises screening, which now focuses on detecting women infected with high-risk types of HPV in many countries. For example, in New Zealand, HPV testing replaced cytology as the primary method of cervical cancer screening in September 2023 (36). Notably, Australia was one of the first countries to implement a national primary HPV screening program, which replaced cytology in December 2017 (37). Some countries without strong histories of vaccination, such as Japan, still use a cytology-based approach to screening. However, studies have suggested that it would be feasible to introduce HPV testing as a primary screening method in Japan (38), and this will happen for women aged 30–60 from 2025 (39). Depending on the triage method chosen, this may increase the demand for colposcopy services, especially in the short term (40). It is therefore essential to enhance the knowledge and skills of physicians specializing in colposcopy. In response, the Japan Society of Gynecologic Oncology has begun initiatives to educate physicians by organizing training sessions on colposcopy (41).

With HPV testing now part of the primary cervical cancer screening approach, colposcopy practices are evolving. Most patients currently requiring colposcopy are those infected with high-risk HPV types, which are most strongly associated with cervical cancer, such as HPV-16 and HPV-18 (42). As vaccination rates increase still further, studies suggest that cases of visible high-grade cervical lesions will decrease, reducing the need for colposcopies (43, 44). However, the situation may be more nuanced than this. For example, a study in Scotland found that when women who persistently test positive following vaccination are positive for HPV subtypes other than HPV-16 and HPV-18, the performance of colposcopy can be affected. This is because lesions associated with subtypes other than HPV-16 and HPV-18 are associated with more subtle changes (45).

The role of colposcopy services is therefore changing. Colposcopists increasingly have a role in estimating diagnoses and defining lesion extent. Specific HPV genotypes identified through screening enable targeted management (46), supporting a more risk-based approach to treatment. However, work from Scotland suggests that, as vaccination coverage increases in different regions, it will probably be necessary to reassess the colposcopy service system and training system to ensure that it remains fit for purpose (44).

There is a need to balance minimizing overtreatment, especially for patients desiring future fertility (47), and ensuring timely interventions to prevent delayed cancer detection. More conservative treatments such as ablation are minimally invasive but carry a risk of treatment failure for high-grade lesions (48). There may therefore be a role for expert colposcopists in advising on the best course of action, taking into account patient preferences. Colposcopists may also directly provide treatment, especially ablation and excision.

There is significant variation in vaccination, screening and treatment practices by region (6, 49). This affects the demand for, purpose of, and training requirements for colposcopy. However, colposcopy services remain an essential part of cervical cancer detection, treatment and prevention around the world. This highlights the crucial importance of the quality assurance of colposcopy, delivered through standards such as those set out in the USA (50) and Europe (51). These standards reflect ideal national or regional practice, but several studies have highlighted the gap between these standards and actual practice (14, 51–54). This suggests that colposcopists in each region or country must take the lead in managing colposcopy services as a whole, including capacity planning. This should consider local and national circumstances to ensure the provision of cost-effective and high-quality colposcopy services.

## 4 Current training for colposcopists

### 4.1 Comparison of current training programs in developed and developing countries

In developed countries, especially the UK, Australia, and New Zealand, structured curricula ensure consistency and reliability in training colposcopists (55). To illustrate the diversity of training systems, **Table 1** presents selected national and regional examples of certification frameworks, while **Table 2** focuses on widely accessible programs, including those from international organizations, that are particularly relevant for low- and middle-income countries. The selections were made to reflect variations in educational structures and to enable comparison across different geographic and institutional contexts. In Europe, the European Federation of Colposcopy has introduced a standard training course using a case-based approach, which has proven effective in multiple countries in improving both skills and confidence, particularly among inexperienced colposcopists (56).

**Table 2:** Aims, content and assessment of training courses on colposcopy.

Course	Aims/objectives	Theoretical element	Practical element	Exam	Ongoing education
BSCCP Colposcopy Training Programme (10)	To ensure the quality of colposcopists and colposcopy services within the NHS Cervical Screening Programme (NHSCSP). To enable trainees to obtain defined core knowledge, and develop the necessary skills and personal and professional attributes to enable competency in colposcopy.	Core knowledge and core competences defined, including both clinical and generic competences. Professional values not detailed but expected to be acquired through practice and supervision.	Trainees are required to carry out at least 150 colposcopies under supervision.	Successful completion of an objective structured clinical examination required for accreditation.	Attendance at one or more BSCCP-approved conferences or meetings every 3 years required to maintain certification.
ASCCP Colposcopy Education Program (34)	None specified.	ASCCP Comprehensive Colposcopy online or in-person course. Knowledge of 2019 Risk Based Management Consensus Guidelines for the Management of Abnormal Cervical Cancer Screening Tests and Cancer Precursors and colposcopy standards recommended.	Hands-on element to in-person courses. Hands-on training and mentorship recommended but not required.	Online exam tests knowledge of ASCCP course, including asking about management of cases using images. Exam certifies completion of course ONLY.	No.
EFC one-day course in basic colposcopy (33)	“The participant knows basic colposcopical methodology and can apply that in a clinical setting”	Lectures on theoretical background of colposcopy, the principles of colposcopy, colposcopic diagnosis and colposcopic management of cases.	Interactive problem-solving sessions including image analysis and case management. No hands-on requirement.	No.	No.
IARC/ IFCPC Distance Learning Colposcopy Course (36)	Train people in low- and middle-income countries to screen HPV-positive women and women with pre-cancerous cervical disease	Online courses covering around 30 topics	15 directly supervised cases (at least 3 high grades), and 50 indirectly supervised cases (at least 10 high grades). Remote possible but trainee must attend clinic at least once.	Objective structured clinical examination.	No.
IGCS Pre-invasive Disease Program (37)	To provide a framework for basic training in pre-invasive disease of the female lower genital tract. To enhance clinical knowledge and skills in pre-invasive cervical disease management for all healthcare providers in any setting.	Two online phases cover theoretical knowledge (basic and advanced). Each phase has five e-learning modules, with a pre- and post-test for each.	Phase 3 is practical hands-on training. An online qualifying assessment must be completed before starting. Mentoring and supervision, and logbook completion required.	Pre- and post-test for each e-learning module. Minimum score required to proceed to next module. Logbook completion, mentor assessment and attendance at an approved course required for completion of Phase 3 and certification.	Potential to set up local Project Echo group as part of continuing clinical practice.

BSCCP, British Society for Colposcopy and Cervical Pathology; ASCCP, American Society for Colposcopy and Cervical Pathology; EFC, European Federation of Colposcopy; IARC/IFCPC, International Agency for Research on Cancer/ International Federation for Cancer Prevention and Colposcopy; IGCS, The International Gynecologic Cancer Society.

In the USA, there is no single certification for colposcopy (50). However, there are many unique training programs available, including one run by the American Society for Colposcopy and Cervical Pathology (ASCCP) (57). Attempts have also been made over many years to define a curriculum for colposcopy training [see, for example (58),]. Studies have also examined different approaches to education and training, such as on-the-job training in humanistic skills at colposcopy clinics (10).

In many developing countries, comprehensive training programs are limited. This has prompted a range of alternatives to be developed. For example, the International Federation for Cancer Prevention and Colposcopy (IFCPC) and International Agency for Research on Cancer (IARC) now offer distance learning courses to support the development of colposcopy skills (59). These courses include theoretical learning, structured training, and evaluation through objective structured clinical examinations (OSCEs). The International Gynecologic Cancer Society (IGCS) also provides an educational program on the course of pre-invasive disease (60) available through the society’s website.

A few studies have examined the potential for different methods of increasing the availability of colposcopy training in developing countries. For example, one study in China examined the use of a digital platform to supplement in-person colposcopy training (61). It found that the use of this platform improved both competence and confidence among colposcopists. The authors suggested that this approach might be rolled out more widely to increase availability of colposcopy training. Another study examined the use of a ‘train the trainers’ program in Vietnam (62). The authors found no difference in knowledge gained by participants in courses run by the original teachers compared with trainers trained through the program. This suggests that this approach might help to increase the availability of training in developing countries.

Beyond these two studies, there is very little recent literature on methods and approaches for training colposcopists. This suggests that a survey and Delphi study might be a helpful way to gather information and develop a consensus about suitable approaches across either developing or developed countries, and at a national or regional level, depending on what is required.

### 4.2 Components of effective training programs

Previous studies suggest that there are two key elements of an effective training program: content or curriculum and delivery method. It seems likely that choices for both will depend on location and resources available. **Table 2** compares the aims, content types, delivery method and certification requirements of five current colposcopy courses (those provided by the BSCCP, ASCCP, EFC, IARC-IFCPC and IGCS). It shows that both online and in-person courses are available, suggesting that choice may depend on both personal preference and access. The table also highlights that courses providing certification have an examination element and usually require a commitment to practical education undertaken over time. This is often proven through the completion of a logbook of competences and mentoring, as well as a practical examination. This suggests that experienced colposcopists will be required to act as mentors and supervise practical training. This might be required on a wider basis, and not just for specific training courses. The role of mentors should therefore be clarified to ensure that enough trained colposcopists are available to fill this role.

In developing either a curriculum or method of delivery, it will be necessary to develop a local or regional consensus on requirements. This might be done through a Delphi study, similar to the one carried out to develop the European Federation of Colposcopy’s core training curriculum for colposcopy (63). This proved to be an effective method for both obtaining information about current training and assessment methods, and reviewing the proposed core curriculum. It might therefore be used as a model elsewhere, including developed and developing countries.

## 5 Addressing the challenges

There are, therefore, several key challenges facing colposcopy services. They include access to trained and suitably qualified personnel, different expectations and demand for colposcopy in different regions and countries, variations in training curricula, and availability of training. Access to colposcopy is also affected by economic disparities between countries and regions, and limitations in the supply of resources. This includes human resources such as doctors and nurses as providers. There are also several ways in which these challenges could be addressed, such as the setting of quality standards for colposcopy practice and/or the training of colposcopists. Alternative approaches to colposcopy practice and training may also help, including the use of remote guidance and AI assistance.

### 5.1 Standards in existing national and regional settings

Some countries have established quality indicators to ensure the quality of colposcopy. For example, in the USA, the American Society for Colposcopy and Cervical Pathology (ASCCP) has developed recommendations for a list of 11 quality indicators for colposcopy (50). These indicators are intended to act as a starting point for quality improvement in colposcopy. As part of their work, the ASCCP working group also carried out a review of international guidelines related to quality improvement (53). They identified 18 documents that contributed to the development of their list of indicators, including reports of working groups, Delphi studies and government guidance. They created four tables summarizing the state of international quality indicators at the time. These tables covered documentation and reporting requirements at the time of colposcopy, the time interval between abnormal screening and colposcopic examination, recommendations and quality indicators related to cervical biopsies and excision procedures, and finally, recommendations and quality indicators related to colposcopist training, certification and maintenance of adequate colposcopist standards.

Six of the 11 agreed ASCCP indicators relate to documentation, and four to time limits for contacting or seeing patients after an abnormal screening result. The final indicator is about taking multiple biopsies targeting all areas with abnormalities (50). The 11 quality indicators proposed by the ASCCP did not include items related to colposcopist training or certification.

Since the ASCCP review was published, further quality indicators or standards for colposcopy have been published in Europe on both essential (64) and expert colposcopy (65). The consensus statement on essential colposcopy (66) provides guidance for general colposcopists seeing women who have been referred following an abnormal screening test. It sets out the aims of colposcopy and explains that it should only be conducted by trained and preferably certified colposcopists, or trainee under supervision. It makes clear that women should be given verbal and written information to facilitate informed consent. It then sets out standards for practice, including management and treatment of various levels of lesion. The statement on expert colposcopy (65) provides guidance on the care options available and the management of challenging cases, often those referred on following standard colposcopy. Both statements make recommendations for standards for care, rather than the indicators that should be used to assess or improve care.

Some additional indicators have been proposed in the USA for endocervical curettage at colposcopy (66). Performance indicators have also been proposed at a population level in Canada (67). These included colposcopy uptake, histologic investigation (biopsy) rate, colposcopy referral rate, failure to attend colposcopy, treatment frequency in women 18–24 years of age, re-treatment proportion, colposcopy exit-test proportion, histologic investigation (biopsy) frequency after low-grade Pap test results, length of colposcopy episode of care, and operating room treatment rate. Two descriptive indicators were also identified: colposcopist volume and number of colposcopists per capita. A later study tested five indicators in Ontario (68). These indicators were also at the population level, and were the percentage of women seen for colposcopy after a first diagnosis of atypical squamous cells of undetermined significance, without evidence of repeat cytology; the median wait time to colposcopy for atypical glandular cells, atypical squamous cells, where high-grade squamous intraepithelial lesions cannot be ruled out, and high-grade squamous intraepithelial lesions; the percentage of women with high-grade Pap test results who were seen in colposcopy within 6 months; the percentage of women who were not seen in follow-up within 12 months after treatment for cervical dysplasia; and the percentage of women who discontinued colposcopy after three normal Pap test results following treatment for cervical dysplasia.

In summary, the work of Mayeaux and colleagues (50, 53), plus the more recent standards and indicators from Europe (64, 65) and Canada (67, 68), provide a strong starting point for any country or region wishing to develop standards or quality indicators to improve the quality of colposcopy services. However, the range of indicators available makes clear that countries and regions need to take into account local variations in practical application and skill levels in developing indicators at both practice and population levels (69). Experience in Ireland has also highlighted the importance of issues like capacity planning in developing cervical cancer screening programs (70) alongside the associated standards and indicators.

**Table 3** below provides a synthesized cross-regional comparison of colposcopy training and certification quality indicators, based on national or regional standards referenced in this section. This comparison highlights both common core components and context-specific variations in practice and implementation.

**Table 3:** Benchmark of quality indicators among countries or regions.

BSCCP	ASCCP	EFC (EU)	NCSP (NZ)	AEPCC (ESP)
Notification of results				
·Within 4 weeks (QI≥90%) ·Within 8 weeks (QI: 100%)	·HSIL: Within 4 weeks ·Suspicion of invasive cancer: Within 2 weeks (60%≤QI ≤ 90%)	·Varies by country (not specified)	·Within 30 working days (QI≥95%)	·Within 8 weeks (QI≥90%)
Interval from cytology to colposcopy				
·HSIL+ or AGC: within 2 weeks (QI≥93%) ·Others: within 6 weeks (QI≥99%)	·HSIL: within 4 weeks ·Suspected invasive cancer: within 2 weeks (60%≤QI ≤ 90%)	·Varies by country (not specified)	·Suspected invasive cancer: within 10 working days ·HSIL or AGC: within 20 business days ·LSIL (including persistent hrHPV-positive): within 26 weeks ·Persistent hrHPV-positive after HSIL treatment (within 3 years): within 4–12 weeks (QI≥95%)	·NILM with hrHPV positivity: within 16 weeks ·ASC-US (hrHPV-positive) or LSIL: within 8 weeks ·ASC-H, HSIL, or AGC: within 4 weeks ·AIS or suspected invasive cancer: within 2 weeks (QI≥80%)
Documentation of findings				
·Reason for referral, hrHPV and cytology findings, TZ type, colposcopic findings of the cervix (including SCJ visibility and cervical visualization), presence of extension into the vaginal wall or endocervical canal, lesion location, colposcopic impression, biopsy site (recommended) (QI: Not numerically specified )	·Visibility of SCJ (QI≥90%) ·Presence of acetowhite epithelium (QI ≥90%) ·Colposcopic impression (QI ≥80%) ·Cervical visibility (QI ≥70%) ·Lesion visibility (QI ≥70%) ·Lesion location (QI: 0–100%)	·Documentation of TZ type in colposcopic assessment (QI: 100%)	·Accurate documentation of colposcopic findings (including SCJ visibility) at initial and subsequent assessments (QI: 100%)	·Documentation of lesion characteristics (type, location, visibility) (QI ≥90%) ·Documentation of colposcopic impression (QI ≥90%)
Time to treatment intervention				
·See and treat (without biopsy) is permitted ·HSIL: within 4 weeks (QI ≥90%), within 8 weeks (QI = 100%)	·See and treat is permitted for high-risk patients aged ≥25 years * ·For patients under 25, follow-up every 6 months is also acceptable (QI: Not specified) * At least 2 of the followings: HSIL cytology, HPV 16 and/or HPV 18 positive, high-grade colposcopy impression	·Not determined	·CIN2/3: within 8 weeks (QI≥90%)	·HSIL/CIN3: within 8 weeks (QI: Not specified)
Treatment				
·Excision: single intact specimen (not fragmented) (QI ≥80%) ·Target excision length:TZ1: >7 mm (but <10 mm in reproductive age) TZ2: 10–15 mm, TZ3: 15–25 mm (QI >95%) ·Pathological appropriateness of excised specimen (QI ≥90%) ·Histological treatment failure within 12 months ≤5% (QI ≤5%)	Not specified	·Colposcopy prior to treatment for screening abnormalities (QI = 100%) ·Negative endocervical margin in excised specimen (QI >80%) ·Documentation of SCJ (QI = 100%) ·Excisional specimens should confirm CIN2 or worse (QI>85%)	·Outpatient LLETZ under local anesthesia (QI ≥80%) ·Women who undergo ablative therapy should have histologic assessment confirming appropriateness (QI = 100%)	·Excision: single intact specimen (not fragmented) (QI ≥80%) ·Excision performed under colposcopic guidance (QI ≥90%) ·Outpatient treatment (QI ≥70%) ·Major hemorrhagic complications requiring treatment (QI <5%) ·Hospitalization due to complications (QI <2%) ·Excision confirms HSIL/CIN2+ (QI ≥70%), no lesion present (QI ≤15%), Overall margin positivity (QI ≤20%), Endocervical margin positivity (QI ≤15%)
Post-treatment follow-up				
·HSIL/CIN2–3: hrHPV testing at 6 months post-treatment → If negative: return to 3-yearly screening (QI = 100%) → If positive: reflex cytology + colposcopy (QI = 100%)	After the initial HPV-based test at 6 months, annual HPV or cotesting is preferred until 3 consecutive negative tests have been obtained.	·Follow-up for up to 20 years after CIN treatment ·hrHPV testing at 6 months (alone or with cytology) ·Repeat cytology and hrHPV at defined intervals, recommend colposcopy if abnormal (QI not specified)	·Women treated for CIN2/3 should receive cytology and colposcopy within 9 months (QI ≥90%)	·Residual or recurrent HSIL at 6 months post-treatment (QI ≤ 10%) ·Residual or recurrent HSIL at 24 months post-treatment (QI ≤ 5%)
Management of non-attenders				
· New patients requiring treatment (QI<10%) ·Missed follow-up appointments (non-attenders) (QI<15%)	·No guideline or QI specified	·Determined individually by each country (no unified QI)	·No-show rate for initial appointments and follow-up (QI<15%)	·No guideline or QI specified

Reference BSCCP (71), ASCCP (50), EFC (51), NCSP (72), AEPCC (73).

BSCCP, British Society for Colposcopy and Cervical Pathology; ASCCP, American Society for Colposcopy and Cervical Pathology; EFC, European Federation of Colposcopy; NCSP(NZ), National Cervical Screening Program (New Zealand); AEPCC, Asociación Española de Patología Cervical y Colposcopia.

### 5.2 Alternative approaches to training or practice

It might be possible to find ways to increase the number of colposcopists, such as new training methods to supplement existing programs. Previous work suggests that possible options might include distance learning (59, 60), the use of digital platforms to supplement in-person training (58) or ‘train the trainer’ schemes (62). These would ideally be coupled with defined standards for training to increase consistency in practice standards for colposcopy. Another possibility might be to expand the range of professional backgrounds from which colposcopists are drawn, and particularly to increase the use of nurse practitioners. Studies have suggested that diagnostic accuracy is comparable between nurses and physicians (15, 16). However, making this change may be challenging in countries such as Japan, where nurse practitioners do not currently provide colposcopy. Elsewhere, it could require culture change to improve the acceptability of provision of services of this kind by non-physicians.

New technology may also provide ways to assist colposcopists. A systematic review of 30 studies on digital colposcopy imaging analysis approaches, and their use in clinical diagnosis, found important benefits from using artificial intelligence (AI), particularly in increasing accuracy (74). AI-based models have also shown promise in increasing the specificity of diagnosis of cervical cancer (75–78). Using a model of this type would therefore mean that fewer cases of the disease would be missed. This is likely to be true even when colposcopy is carried out by relatively inexperienced staff (79). This approach could therefore be particularly useful in low- and middle-income countries, where experienced colposcopists may be unavailable (76). Both expanding the professional backgrounds from which colposcopists are drawn and integrating AI technology into colposcopy practice would have implications for the training of colposcopists. These approaches would therefore need a review of current training to ensure that it was fit for purpose.

## 6 Future directions

This review has identified several gaps in the existing research on the role of colposcopists and colposcopy services in cervical cancer prevention, diagnosis and treatment. These provide a useful framework for future work in this area.

The first area is building a consensus on the skills and knowledge required of colposcopists and developing knowledge of the current training available. This might use a Delphi study, such as the work carried out in Europe (63) to develop a consensus on the training requirements for colposcopists. This approach could also usefully identify what is currently in place on a local or regional basis, as well as develop a consensus on training needs in a particular geography or area. For example, Japan, the location that we know best, has some specific challenges related to the low levels of HPV vaccination and the convention that colposcopy is carried out by physicians. The training needs there may therefore be quite different from those in somewhere like the UK, where nurse colposcopists are common, and there is a strong HPV vaccination program.

The second area is the application of global standards at local or regional levels. Researchers might look at how regional standards are applied at the national level, for example, in Europe (80). This could provide insights into how global standards might be implemented more locally. It might also be fruitful to look at how existing global standards in healthcare are applied regionally and nationally. This might include examining how guidelines issued by the WHO, including on cervical cancer screening (81), are interpreted and implemented.

The third area is to explore training for soft skills such as communication and anxiety reduction. There is very little research on how colposcopists can develop these skills, despite widespread agreement that they are important. The fourth area relates to the use of AI to support diagnosis via colposcopy. There is little doubt that there is potential for this application of AI, but few studies have examined the likely effect on the training requirements for colposcopists, or how it might affect how training is delivered.

Finally, colposcopy services should not exist in isolation but should be integrated as part of an organized clinical pathway. This may require further studies and discussion on the clinical governance of these services, including from the perspectives of hospitals, health authorities, and policymakers.

## 7 Conclusion

This review has highlighted that the requirements for colposcopy vary considerably by region. Areas with higher and lower incidences of cervical cancer have different needs. The patients requiring colposcopy also vary depending on factors such as the national level of HPV vaccination and the primary screening approach. However, until the widespread adoption of vaccines leads to a significant decline in the incidence of cervical cancer, the identification of at-risk patients through colposcopy, and their management based on established guidelines, remain crucial for the elimination of cervical cancer. Colposcopy without assured quality can provide a false sense of security, which is harmful. It may also lead to overtreatment, where patients who do not actually require surgery are subjected to unnecessary procedures, causing further harm. The decision about the appropriate treatment is best made by highly skilled colposcopists.

There is unmistakably a correlation between high levels of cervical cancer and poor access to resources such as colposcopy. It may therefore be appropriate to standardize colposcopy practices at the national level. This will also allow new technologies to be introduced to address national needs. The decision about what to include in colposcopists’ training, especially advanced or expert training, is also probably best left at the national level. However, the experiences of both the IARC-IFCPC and IGCS suggest that it may be possible to develop a basic standard curriculum for colposcopist training at a global level. This will provide assurance that a basic minimum standard has been met, but countries will be able to consider how they can best incorporate aspects that are culture-dependent, such as colposcopists’ social roles in patient education.

In summary, colposcopists and colposcopy services play an important role in the global effort to reduce the incidence of cervical cancer. This role varies between countries, depending on factors such as the level of HPV vaccination and testing, the resources available, and access to healthcare. However, the training required for colposcopists seems relatively consistent across settings. New technologies offer potential for addressing some of these access issues, but countries must be free to adapt practice and training standards to meet national circumstances to ensure that their colposcopy services are fit for purpose at a national level.
